# A meta-review of transparency and reproducibility-related reporting practices in published meta-analyses on clinical psychological interventions (2000–2020)

**DOI:** 10.3758/s13428-021-01644-z

**Published:** 2021-06-26

**Authors:** Rubén López-Nicolás, José Antonio López-López, María Rubio-Aparicio, Julio Sánchez-Meca

**Affiliations:** 1 Facultad de Psicología, Campus de Espinardo, Universidad de Murcia, edificio n° 31, 30100 Murcia, Spain; 2 Universidad de Alicante, Alicante, Spain

**Keywords:** Meta-analysis, Reproducibility, Transparency and openness practices, Meta-science, Data sharing

## Abstract

Meta-analysis is a powerful and important tool to synthesize the literature about a research topic. Like other kinds of research, meta-analyses must be reproducible to be compliant with the principles of the scientific method. Furthermore, reproducible meta-analyses can be easily updated with new data and reanalysed applying new and more refined analysis techniques. We attempted to empirically assess the prevalence of transparency and reproducibility-related reporting practices in published meta-analyses from clinical psychology by examining a random sample of 100 meta-analyses. Our purpose was to identify the key points that could be improved, with the aim of providing some recommendations for carrying out reproducible meta-analyses. We conducted a meta-review of meta-analyses of psychological interventions published between 2000 and 2020. We searched PubMed, PsycInfo and Web of Science databases. A structured coding form to assess transparency indicators was created based on previous studies and existing meta-analysis guidelines. We found major issues concerning: completely reproducible search procedures report, specification of the exact method to compute effect sizes, choice of weighting factors and estimators, lack of availability of the raw statistics used to compute the effect size and of interoperability of available data, and practically total absence of analysis script code sharing. Based on our findings, we conclude with recommendations intended to improve the transparency, openness, and reproducibility-related reporting practices of meta-analyses in clinical psychology and related areas.

Systematic reviews and meta-analyses are commonly ranked among the most relevant sources of scientific evidence on the effectiveness of healthcare interventions (Evans, 2003), and therefore provide a powerful tool to evidence-based healthcare practice. Importantly, the validity of the conclusions drawn from a meta-analysis depends on the methodological quality and rigor of the primary studies (Nuijten et al., 2015; van Assen et al., 2015).

The past decade has revealed significant problems in terms of replicability and reproducibility in psychological research, leading to the so-called replication crisis (McNutt, 2014; Open Science Collaboration, 2015; Pashler & Wagenmakers, 2012). In this paper, by ‘replicability’, we mean that a previous conclusion will be supported by novel studies that address the same question with new data, and by ‘reproducibility’, we refer to obtaining the exact same previous result applying the same statistical analysis to the same data (Asendorpf et al., 2013; Epskamp, 2019).

Several efforts have been made to evaluate the replicability of findings from psychology and related fields (e.g., Hagger et al., 2016; Klein et al., 2014; Open Science Collaboration, 2015). A number of methodological issues, questionable research practices, and reporting biases have been suggested as potential explanations for failed replication attempts (Ioannidis, 2005; Johnson et al., 2017; Schmidt & Oh, 2016; Simmons et al., 2011; Stanley et al., 2018; Szucs & Ioannidis, 2017). In this context, meta-research has emerged as an approach ‘to investigate quality, bias, and efficiency as research unfolds in a complex and evolving scientific ecosystem’ (Hardwicke, Serghiou, et al., 2020a, p. 12; Ioannidis, 2018). This ‘research on research’ aims to help identify the key points that could be improved in research and reporting practices.

Different concerns about the reproducibility of published meta-analyses have also emerged. Gøtzsche et al. (2007) recomputed the primary effect sizes from 27 meta-analyses, finding problems in 10 of them. Tendal et al. (2009) recomputed the primary effect sizes and summary meta-analytic estimates re-extracting the relevant primary statistics by independent coders, finding substantial inconsistencies. In a similar way, Tendal et al. (2011) found that multiplicity of effect sizes in primary studies can lead to different meta-analytic conclusions depending on how such multiplicity is addressed. Lakens et al. (2017) struggled to reproduce a set of meta-analyses due to lack of access to raw data and incomplete reporting of the methodology followed. Kvarven et al. (2020) compared the results of published meta-analyses to large-scale replications on the same topic, finding significant differences in effect sizes for 12 out of the 15 pairs. And last, Maassen et al. (2020) found a number of challenges in reproducing the calculation of effect sizes based on the information reported by the original authors of each meta-analysis.

Of note, carrying out a meta-analysis involves a multi-decision process from the literature search to the statistical analysis, and only if such decisions are clearly stated will the meta-analysis be reproducible by an independent research team. Open science initiatives are a major point here: preregistration, sharing open material and data, and sharing open analysis scripts offer several benefits (Federer et al., 2018; Hardwicke & Ioannidis, 2018b; Nelson et al., 2018; Nosek et al., 2015; Nosek et al., 2019; Nosek & Lindsay, 2018; Popkin, 2019). The importance of promoting and adopting open science practices in meta-analysis has been increasingly recognized in recent years (Lakens et al., 2016; Moreau & Gamble, 2020; Pigott & Polanin, 2020). For instance, preregistered meta-analyses avoid to some extent practices such as selective inclusion or reporting of results (Page et al., 2013). Additionally, open meta-analytic data sharing offers several benefits related to efficiency in scientific development and reproducibility or robustness checking. Full, machine-readable availability of meta-analytic data allows for easy updating, reusability for new purposes, reanalysis with different or novel analysis techniques, and quick checking of possible errors. Along with the availability of meta-analytic data, open script code sharing allows for easy analytic reproducibility checking and involves a straightforward statement of the analytic methods applied. All these points are particularly relevant in the context of meta-analysis, given that meta-analysis claims may have a strong impact on policymaking or healthcare practices. In addition, meta-analyses should keep the results updated as new primary evidence emerges. It is important to note that there is no single perspective concerning which analytic methods should be applied in meta-analysis, so that novel analytic methods are regularly being developed. Applying such novel techniques to published data could be enlightening.

The last years have seen a proliferation of reviews assessing the prevalence of transparency and reproducibility-related practices in primary studies. A common finding across such reviews is the lack of transparency in the reporting of key indicators for reproducibility. Some of these reviews examined broad research disciplines such as biomedical sciences (Iqbal et al., 2016; Wallach et al., 2018), social sciences (Hardwicke, Wallach, et al., 2020c), and psychology (Hardwicke, Thibault, et al., 2020b). In the meta-analytic arena, Polanin et al. (2020) assessed the compliance with transparency and reproducibility-related practices of all meta-analyses published in *Psychological Bulletin*, finding poor adherence to these guidelines. This restriction to a specific journal arguably yielded a pool of high-quality meta-analyses, but it remains unclear whether the patterns observed can be generalized to other journals with different editorial guidelines and requirements. While Polanin et al.’s (2020) approach provides an overview of the reporting quality of meta-analyses across a wide range of scientific topics, it also makes it difficult to characterize the reporting pattern in a specific research area.

## Purpose

In this study we empirically assessed the prevalence of transparency and reproducibility-related practices in published meta-analyses on clinical psychological interventions examining a random sample of 100 meta-analyses. Our purpose was to identify the key points that could be improved in the field of clinical psychology and to produce some recommendations accordingly. We selected the area of effectiveness of clinical psychological interventions for three main reasons. First, we intended to offer recommendations focused on a specific research topic, since transparency and openness practices might vary across research areas. Second, meta-analysis on the effectiveness of clinical psychological interventions is one of the types of meta-analysis most frequently published in psychological research. Third, meta-analyses on the effectiveness of clinical psychological interventions have an important impact on clinical practice and policymaking.

## Method

### Design

This is a meta-review, that is, a kind of umbrella review that can be defined as a methodological systematic review of meta-analyses (Biondi-Zoccai, 2016).

### Identification and selection of studies

Published meta-analyses of clinical psychological interventions were identified conducting a systematic electronic search in PubMed, Scopus, and the core collection of Web of Science. The search was carried out on 22 January 2020. The full search strategies followed in each database are available in Supplementary file 1: https://osf.io/z5vrn/. Articles were included if the following criteria were met: (a) at least one meta-analysis focused on the effectiveness of psychological intervention/s was reported; (b) publication year after 1999; (c) the effect size index was a mean difference or a standardized mean difference; and (d) written in English or Spanish. Individual participant data meta-analyses and network meta-analyses were excluded from this study.

All records identified by the electronic search were downloaded in bibliographic format and duplicates were removed using the R package ‘*revtools’* (Westgate, 2019), first by exact match from DOIs, and subsequently by fuzzy matching from titles. All bibliographic files (the outputs of electronic search and the output of unique references) and the script code used to remove duplicates are available at: https://osf.io/xg97b/. Unique references were uploaded to the open-source program ‘*abstrackr’* (Wallace et al., 2012) for the screening. The titles and abstracts of the unique references were assessed by one author (RLN), and references that were clearly ineligible were excluded at this stage. When the information presented in title and abstract was insufficient, the full-text records were evaluated independently by two authors (RLN and MRA), with a third author (JSM or JLL) getting involved to resolve any disagreements. Supplementary file 1 available at: https://osf.io/z5vrn/ presents a flow chart summarizing the screening process.

### Sampling

A total of 664 meta-analyses were identified by the electronic search and screening process. Of these, 100 were randomly selected using a random number generator between 1 and the total number of meta-analyses included, setting up a certain seed to guarantee the reproducibility of the process. Supplementary file 1 available at: https://www.osf.io/z5vrn/ presents two overlapping histograms displaying the distribution of the year of publication for the included meta-analyses and for the selected random sample. In order to compare the two observed distributions, the Kolmogorov–Smirnov test was performed. Equivalence was found between both distributions (*D =* .104, *p =* .299)*.*

### Procedure and data extraction

A structured coding form was created based on previous studies (Hardwicke, Wallach, et al., 2020c; Iqbal et al., 2016; Koffel & Rethlefsen, 2016; Wallach et al., 2018) and existing meta-analyses guidelines (Liberati et al., 2009; Pigott & Polanin, 2020). The coding form is available at: https://www.osf.io/2dzmk/.

Items were grouped into nine different categories: (a) study ID and study characteristics (items 1-7); (b) preregistration, protocol, and the statement of compliance with guidelines (items 7–13); (c) identification and selection of studies (items 14–23); (d) data collection process (items 24–29); (e) effect or summary measures (items 30–35); (f) statistical methods (items 36–46); (g) data and script analysis availability (items 47–59); (h) conflict of interest and funding statement (items 60–61); and (i) access format of the paper (item 62).

At a first stage, the coding form items were tested in a pilot coding. Four authors (RLN, MRA, JSM and JLL) independently applied the coding form to a random sample of five meta-analyses. Subsequently, in a series of meetings, disagreements between the coders were resolved by discussion until consensus was reached. During this process, items were modified or refined where necessary.

Next, two authors (RLN and MRA) independently applied the coding form to the 100 meta-analyses randomly selected. The coding form was applied between 3 April and 29 May 2020. Discrepancies between the two coders were resolved by discussion and review of the relevant materials. The three data sets (coder 1, coder 2, and consensus data) are available at: https://osf.io/xg97b/. Inter-coder agreement was assessed with Cohen’s kappa coefficient, for close-ended items, using the R package *‘irr’* (Gamer et al., 2019). The resulting values ranged between .55 and 1, with only two items yielding values below .6 (item 16 and 55, see Supplementary file 2 available at: https://osf.io/tw6cd/).

In addition, the format used to share each kind of raw data available was coded a posteriori^1^*,* given the implications of this aspect for the efficient reusability of the data. Thus, six sub-items paired with items 50–55 were added. The formats were categorized as interoperable or not (Bek, 2019; Wilkinson et al., 2016) based on two criteria: format that allows easy manipulation and reading of the values for open-source statistical software, and proprietary/non-proprietary format.

### Analysis

First, we examined how often each of the indicators was reported across meta-analyses. For each proportion, we calculated 95% confidence intervals based on the Wilson score interval (Wilson, 1927) for binomial items and on the Sison–Glaz method (Sison & Glaz, 1995) for multinomial items, using the R package *‘DescTools’* (Signorell et al., 2020).

Furthermore, we explored possible associations using binary logistic regression, with publication year (item 4), preregistration (item 7), and use of reporting guidelines (item 12) as predictors, and the following dichotomous (or dichotomized by removing the ‘Other’ category) indicators as dependent variables: items 15 to 20, 22 to 32, 34, 36, 38 to 42, 44, and 50 to 55. We started fitting single predictor models to observe unadjusted associations, and then switched to multiple regression models introducing all three predictors to explore the associations for each predictor controlling for the others. We quantified the strength of the associations by calculating odds ratios and 95% confidence intervals based on profile likelihood. Despite the large number of contrasts performed, we did not introduce any corrections for multiple comparisons due to the exploratory nature of our analyses.

Preparation of data and all figures presented in this paper was accomplished using the collection of R packages *‘tidyverse’* (Wickham et al., 2019). All the script codes used to handle and analyse the data are openly available at: https://osf.io/xg97b/.

## Results

The total of 664 included meta-analyses were published between 2000 and 2020 (median = 2015), whereas publication year for the selected random sample of 100 meta-analyses ranged between 2001 and 2020 (median = 2016).

### Preregistration, guidelines, and conflict of interest

**Fig. 1:**
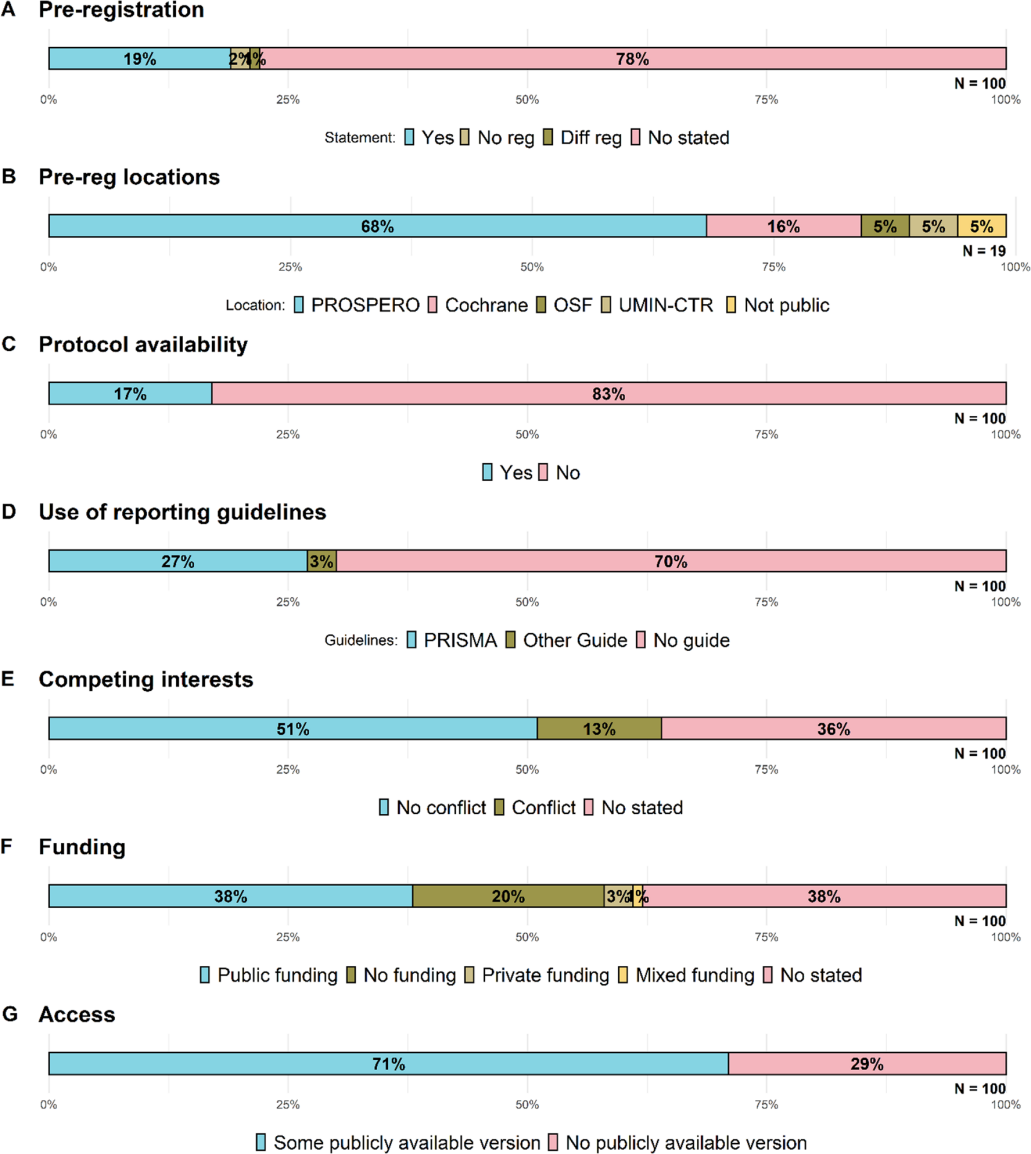
Percentage of b meta-analysis preregistered, **b** preregistration locations, **c** protocol availibility, **d** guidelines adherence, **e** competing interest statements, **f** funding statements, and **g** accesibility of meta-analyses. *N* indicates total number of meta-analyses assessed for each indicator

Of the 100 meta-analyses examined, 19 (see Fig. 1a) stated that there was a preregistration of the study; of these, 13 (68%, Fig. 1b) allocated their preregistration in PROSPERO, three (16%) in the Cochrane Library, one (5%) in OSF, one (5%) in UMIN-CTR, and one (5%) internally at a national agency. Conversely, 78 out of the 100 meta-analyses in our random sample did not include any statement on preregistration, whereas two stated that there was no preregistration and one mentioned preregistration of a different project. Only 17 out of the 100 meta-analyses included a link or a unique ID to locate an accessible protocol (Fig. 1c).

With regard to the statement of compliance to guidelines (Fig. 1d), 70 out of the 100 meta-analyses did not mention following any reporting guideline, whereas 27 stated that they followed PRISMA and three that they followed other guidelines (MARS in two studies and QUOROM in one).

Funding sources and competing interests could be a potential source of bias. Of the 100 meta-analyses reviewed, 13 (see Fig. 1e) stated one or more conflicting interests, 51 stated that there were no conflicting interests, and 36 did not include a conflict of interest statement. With regard to funding, 38 meta-analyses (see Fig. 1f) failed to include a funding statement, whereas 38 declared public funding sources, three mentioned private sources, one declared both public and private sources, and 20 stated that no funding was provided. Regarding accessibility, 29 of the 100 meta-analyses had no publicly available version; of these, 13 stated that public funding was provided.

### Systematic review methods

#### Eligibility criteria and literature search

**Fig. 2:**
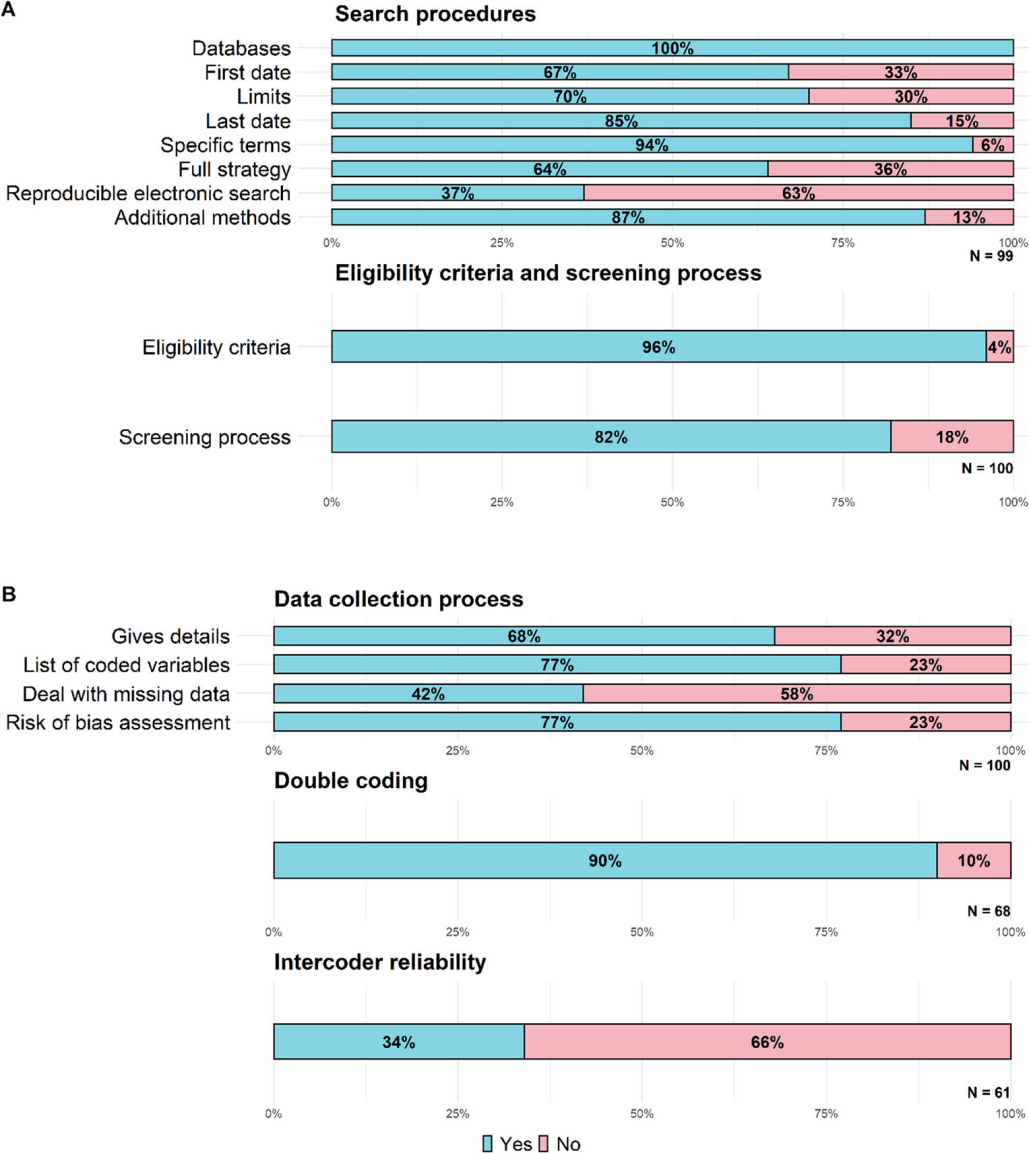
Percentage reported of systematic review methods by **a** eligibility criteria and literature search, and **b** data collecion process, showing different indicators for each category. *N* indicates total number of meta-analyses assessed for each indicator

Detailed and complete reporting of the search and screening procedures allows the assessment of the quality of the procedure and facilitates replication. We excluded one meta-analysis because it consisted of a reanalysis of a previous meta-analysis. Thus, this meta-analysis was excluded from the analysis of the items concerning electronic search (items 14 to 20). All the remaining 99 meta-analyses specified the electronic databases consulted (Fig. 2a); of these, 66 (67%) specified the year for first date searched (including database inception); 69 (70%) indicated the electronic search limits used; 84 (85%) specified the month and year of the electronic search; 93 (94%) included the search terms used; and 63 (64%) reported the full search strategy (exact terms and the Boolean connectors). However, only 37 reported all these details combined, which is required for the electronic search to be completely reproducible; 86 (87%) declared having used additional search methods as follows: 78 (91%) used additional backward searches of reference lists of identified articles or relevant previous reviews, 29 (34%) used additional hand searches of relevant websites, conferences papers, relevant journals, etc., 23 (27%) contacted experts, nine (10%) consulted Google Scholar, and five (6%) used additional forward searches by citation tracking.

Among the 100 meta-analyses examined, 96 (Fig. 2a) specified the eligibility criteria and 82 described the screening process.

#### Data collection process

The data collection process should be detailed, including the methods for dealing with missing data and for assessing risk of bias in the included studies, so that the accuracy of the extracted data and their validity can be evaluated. Of the 100 meta-analyses, 68 (see Fig. 2b) described details about the collection process of study characteristics; out of these, 61 (90%) conducted double coding, of which 21 (34%) reported inter-coder agreement values. Also, 77 out of the 100 meta-analyses listed all variables for which data were sought, 42 described at least one method to deal with missing data (such as statistical imputation, request to authors), and 77 described methods to assess risk of bias in included studies.

### Meta-analysis methods

#### Effect measures

**Fig. 3:**
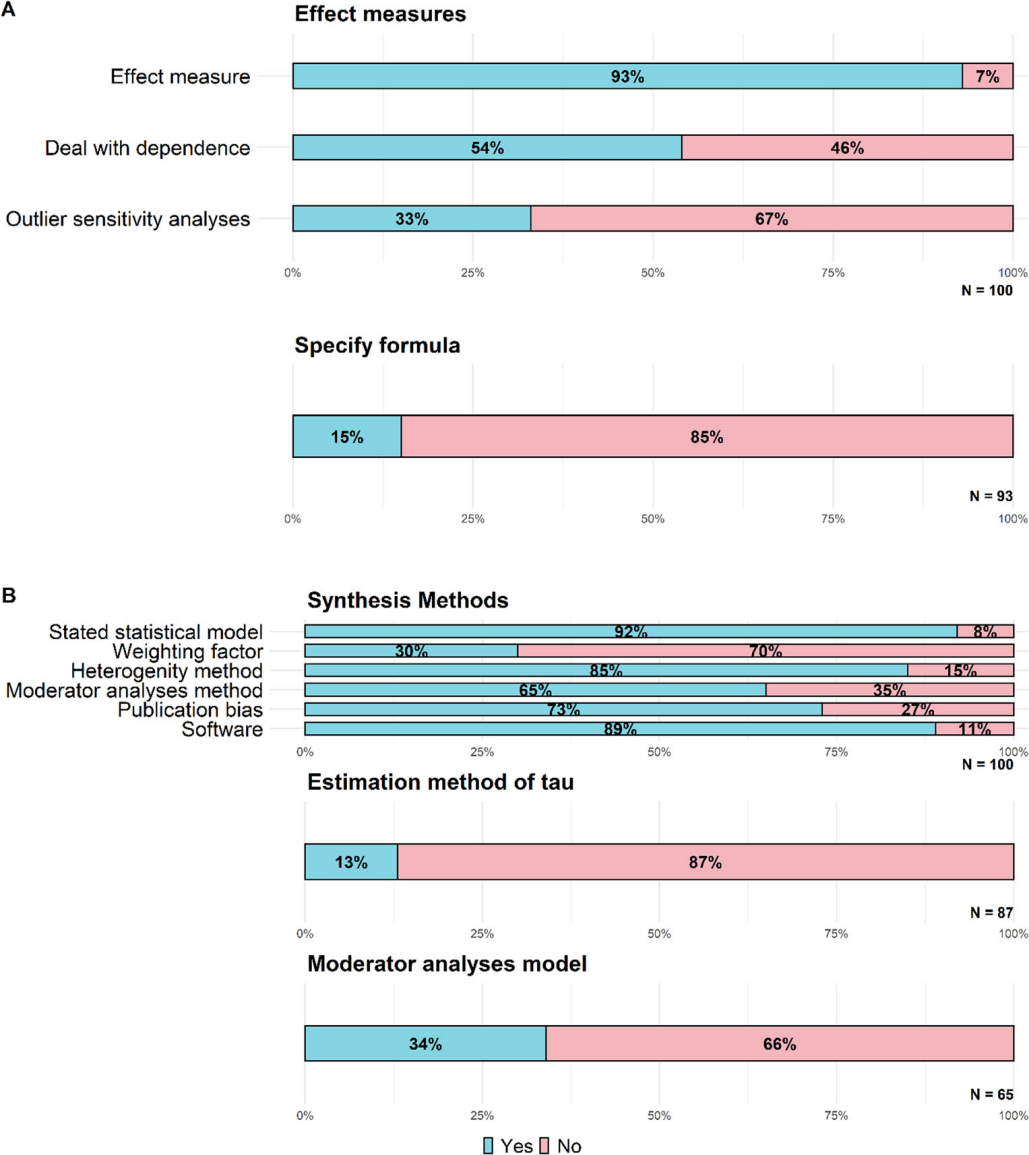
Percentage reported of meta-analysis methods by **a** effect measures, and **b** synthesis and analysis methods, showing different indicators for each category. *N* indicates total number of meta-analyses assessed for each indicator

Identifying the effect measure used and specifying the method to calculate it is crucial due to the existence of many different effect size measures as well as several approaches to calculate some of them (Hoyt & Del Re, 2018; Rubio-Aparicio et al., 2018). The majority of the 100 meta-analyses reported the effect measure used in the synthesis (93% see Fig. 3a); however, the majority of these did not specify in detail which formula was used to compute it (85%).

Multiplicity of results in trial reports leads to statistical dependency if the multiple effect estimates from the same study are based (at least partially) on the same participants, and ignoring it may result in underestimation of standard errors and erroneous statistical conclusions (Bender et al., 2008; López-López et al., 2018; Tendal et al., 2011). About half of the meta-analyses (54%) described at least one method to deal with multiplicity, including random selection, averaging, decision rules, or using advanced meta-analytic methods to model or account for it (López-López et al., 2018). About a third (33%) of the meta-analyses described sensitivity analyses to assess the effect of outliers.

#### Synthesis and analysis methods

The choice of statistical model and meta-analytic method may have an impact on the results and conclusions, hence the importance of reporting a detailed description of the statistical analysis approach (Langan et al., 2015; Sánchez-Meca et al., 2013; Schmidt et al., 2009). The vast majority of the 100 meta-analyses stated the statistical model assumed for the synthesis process (92%, see Fig. 3b), with most of them assuming a random-effects model (87, 95%); however, very few of those meta-analyses stated the estimation method of the heterogeneity variance, τ^2^ (11, 13%). Furthermore, of the total of 100 meta-analyses, only 30 stated the weighting factor used, whereas 85 mentioned methods to assess heterogeneity. Moreover, 65 meta-analyses described methods to assess the influence of possible moderator variables, but only 22 of these (34%) specified the statistical model assumed for the moderator analyses.

Additionally, 73 out of the 100 meta-analyses stated having used at least one method to assess reporting biases (including publication bias); of these, 61 (84%) reported a funnel plot, 34 (47%) applied the trim-and-fill method, 31 (42%) used the Egger test, 24 (33%) applied some form of the fail-safe-N method, 13 (18%) used the Begg and Mazumdar test, and only one used PET-PEESE and p-uniform methods.

Most meta-analyses identified the software used to carry out the statistical analyses (89%); of these, 38 (43%) used Comprehensive Meta-Analysis, 24 (27%) used Review Manager, 20 (22%) used STATA, 12 (13%) used R, eight (9%) used SPSS, and six (7%) used other software.

### Data and analysis script availability

**Fig. 4:**
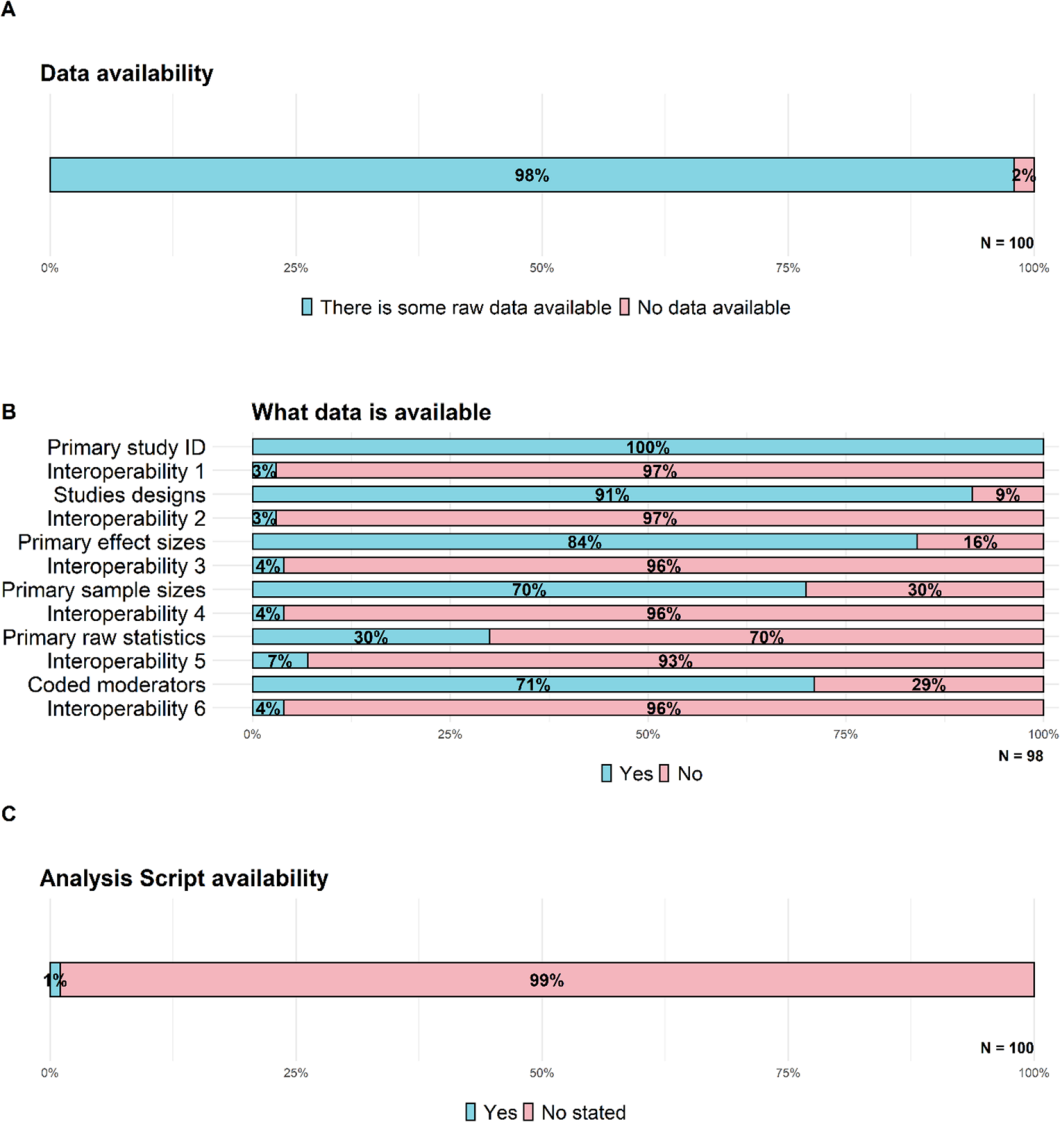
Percentage of **a** meta-analysis that reported some raw data, **c** meta-analysis that shared the analysis script code, and **b** what data were available and if these were in interoperable formats; each interoperability bar corresponds to the primary data represented over it. *N* indicates total number of meta-analyses assessed for each indicator

The unit of analysis of a meta-analysis is usually the primary study, so when we talk about data availability, we typically refer to the summary-level data (e.g., effect sizes) from each primary study included in each meta-analysis. In systematic reviews and meta-analyses, it is common to report the characteristics of the included studies, as well as through table or forest plots, the individual effects measures. The vast majority of the meta-analyses we examined (98%, see Fig. 4a) reported at least some raw data; of these, 93 reported some raw data in the paper itself. Furthermore, 31 meta-analyses included raw data in supplementary files or appendices, four stated that some raw data were available upon request, one shared data using an institutional webpage, and one using https://osf.io/.

Of the 98 meta-analyses for which some raw data were available, all the meta-analyses (see Fig. 4b) identified the primary study associated with the data, only three in interoperable format; 89 reported the primary study comparator (e.g., treatment-as-usual, waitlist, other intervention…), only three in interoperable format; 82 reported the primary effect sizes combined, only three in interoperable format: 69 reported the sample sizes of the groups compared in the primary studies, only three in interoperable format: 29 reported the statistics used to compute primary effect sizes, only two in interoperable format: and 70 reported the coded moderator variables, only three in interoperable format

Data script availability refers to detailed step-by-step descriptions of the analyses carried out (e.g., SPSS syntax, R code etc.). Availability of the analysis code, along with the data shared, enables to check computational reproducibility of the reported results. Unfortunately, only one of the meta-analyses we examined (see Fig. 4c) mentioned that the analysis script code was available (through an OSF link).

### Associations between year, preregistration or guidelines adherence and transparency and reproducibility-related reporting

Several logistic regression models were fitted; for space-saving reasons only a selection of the results is presented in this section. The full results are available at: https://osf.io/9xsg2/

Table 1 presents the odds ratio and 95% CI of the main results of simple and multiple models. Taking into consideration the results of the simple and multiple models, publication year was a significant predictor of the inclusion of a description of the screening process (*OR =* 1.29 [95% CI: 1.12-1.54], the statistical model assumed (*OR =* 1.29 [95% CI: 1.08-1.60]), the methods to assess reporting biases (*OR = 1*.19 [95% CI: 1.06-1.35]), and the software used (*OR =* 1.19 [95% CI: 1.04-1.39]), with more recent studies providing a more detailed description of the methods used. Moreover, preregistered meta-analyses were more likely to specify the year for first date searched (*OR =* 13.27 [95% CI: 2.32-253.59]) and following reporting guidelines such as PRISMA was associated with a more complete report of the full search strategy (*OR =* 3.20 [95% CI: 1.07-11.08]) and the methods used for assessing risk of bias of the individual studies (*OR =* 6.50 [95% CI: 1.12-124.12]).

**Table 1:** Odds ratios and 95% CI between predictors and transparency and reproducibility-related indicators

Indicator	Year		Preregistration		Guideline adherence statement	
	Simple	Multiple	Simple	Multiple	Simple	Multiple
Specify the year for first date searched	1.06 [0.96–1.17]	1.04 [0.93–1.17]	**12.00 [2.30–221.22]**	**13.27 [2.32–253.59]**	1.24 [0.50–3.25]	.64 [0.21–1.93]
Report the full search strategy	1.1 [1.00–1.22]	1.05 [0.94–1.17]	2.50 [0.82–9.38]	1.41 [0.40–5.76]	**4.08 [1.49–13.20]**	**3.20 [1.07–11.08]**
Specify the eligibility criteria operatively	**1.23 [1.01–1.52]**	1.19 [0.95–1.50]				
Describe the screening process	**1.32 [1.16–1.53]**	**1.29 [1.12–1.54]**			**9.30 [1.77–171.82]**	2.44 [0.37–48.35]
List all variables for which data were sought	**1.15 [1.03–1.29]**	1.12 [0.99–1.26]	2.97 [0.90–13.55]	1.56 [0.33–11.25]	**3.60 [1.11–16.25]**	2.27 [0.61–11.18]
Describe methods used for assessing risk of bias of individual studies	**1.17 [1.05–1.32]**	1.10 [0.97–1.25]			**13.29 [2.57–244.28]**	**6.50 [1.12–124.12]**
Identify the statistical model assumed	**1.23 [1.06–1.45]**	**1.29 [1.08–1.60]**			1.31 [0.28–9.34]	0.25 [0.03–2.29]
Identify the estimation method of τ^2^	1.15 [0.96–1.47]	1.06 [0.89–1.34]	**4.55 [1.16–17.42]**	3.12 [0.71–13.41]	3.18 [0.88–12.03]	1.97 [0.47–8.43]
Describe any methods to assess reporting biases (including publication bias)	**1.16 [1.04–1.29]**	**1.19 [1.06–1.35]**	3.79 [0.99–25.08]	4.73 [0.97–38.21]	0.81 [0.32–2.14]	**0.29 [0.09–0.94]**
Mention the software used to carry out the statistical analyses	**1.20 [1.05–1.38]**	**1.19 [1.04–1.39]**	2.54 [0.44–48.04]	1.58 [0.19–35.96]	2.07 [0.49–14.13]	0.99 [0.17–8.25]
Statistics used to compute the effect are size available	1.09 [0.98–1.24]	1.05 [0.93–1.2]	2.08 [0.72–5.86]	1.38 [0.43–4.26]	**2.58 [1.03–6.48]**	2.04 [0.74–5.66]

Odds ratio and CIs not interpretable due to separation were omitted. Odds ratio 95% CI is presented in brackets. Bolded values indicated CIs that do not contain the null value

### Key points

The key points identified where a substantial lack of transparency was found concerning the potential reproducibility of the meta-analyses examined are summarized in Table 2. Other aspects related to the promoting transparency (i.e., well-stablished reporting guidelines adherence) and to the prevention of result-based bias (i.e., preregistration) are summarized in Table 3.

**Table 2:** Summary of results and recommendations on the key points lacking transparency

Point	Reporting rate	Why is it important?	Recommendations
Completely reproducible electronic search	37% [28%–47%]	Facilitates the evaluation of the comprehensiveness of the review and its update in the same direction.	Always report the full search strategy for ALL databases consulted, detailing dates, limits, specific terms, and the Boolean connectors. For space-saving reasons, it is recommended to report these details as supplementary material hosted by the journal or online repositories.
Specify effect measure formula	15% [9%–24%]	Due to the variety of approaches to define standardized and unstandardized mean differences, specification of the formula used is required to ensure the reproducibility of results.	Always report the specific formula on the paper itself or refer readers to a reference (including the equation number and/or the book/article page where the formula can be found).
Identify the weighting factor	30% [22%–40%]	Although inverse variances are the most popular weighting scheme, other alternatives are available, and the choice can have an impact on the results.	Always specify the weighting factor used. Note that this should only take a few words.
Identify the estimation method of the heterogeneity variance, τ^2^	13% [7%–21%]	The between-studies (or heterogeneity) variance is used in random-effects weights and prediction intervals, as well as in the calculation of popular indices in meta-analysis such as I^2^ and pseudo-R^2^. Many estimators of τ^2^ have been proposed, and the resulting estimates often show important discrepancies among estimators.	Always report and justify the estimation method of the heterogeneity variance. The choice should be based on the data set features along with recommendations from simulation studies under conditions similar to those of the meta-analytic database.
Open availability of statistics used to compute the effect size	30% [21%–39%]	This is the primary raw data used to calculate the effect measures. Availability of this information, along with the effect measure formula, allows the analytic reproducibility of primary effect measures.	Always share ALL coded raw data prior to any data handling in easily computer-readable formats, such as *tsv* or *csv.* To facilitate error checking, add a column indicating the precise location of the coded data in the primary study. Online repositories are very useful for this (OSF, Fighshare, Zenodo, GitHub…), but other options include journal or personal websites.
Interoperability of data sharing format	3% [1–9%] 3% [1–9%] 4% [1–10%] 4% [1–12%] 7% [2–22%] 4% [1–12%]	Significantly increases the efficiency of data reusability through the use of computer-readable and non-proprietary value formats. Avoiding the error-prone process of manual recoding of available data for reproduction or reuse attempts.	Always share data in interoperable formats such as *csv or tsv.* The FAIR principles (Wilkinson et al., 2016) are a useful guideline for best practices in data sharing.
Open availability of analysis script code	1% [0–5%]	It contains a detailed step-by-step description of the analyses performed. Sharing it is the best way to ensure the analytic reproducibility and to avoid the ambiguities of verbal descriptions.	Always share the analysis script code. Moreau and Gamble (2020) share a very useful script template for carrying out a meta-analysis with R using the metafor (Viechtbauer, 2010) package in their OSF project: https://osf.io/5nk92/. Again, online repositories, own websites or journal hosting are very useful for hosting the files.

95% CIs are presented in brackets

**Table 3:** Summary of results and recommendations on different practices related to promoting transparency

Point	Practice rate	Why is it important?	Recommendations
Use of reporting guidelines	30% [20–40%]	It’s a very helpful tool that facilitates the transparent reporting of all relevant points on the rationale, methods and results of a systematic review or meta-analysis. Furthermore, it standardizes the report, facilitating the readability, assessment and update of the systematic review and/or meta-analysis.	Use well-established, up-to-date reporting guidelines intended for meta-analyses such as: the recently updated PRISMA 2020 (Page et al., 2021); the focused-on reliability generalization meta-analyses REGEMA (Sánchez-Meca et al., 2021); the focused-on non-intervention studies NIRO-SR (Topor et al., 2020), for example.
Preregistration	19% [12–17%]	It prevents the result-based bias by stating the main hypotheses, design and analysis plans prior to obtaining the results. Furthermore, it could provide a transparent project timeline, workflow and general decision-making process.	Specialized repositories such as PROSPERO could be helpful since they are tailored to the SR/MA design. General repositories such as OSF could also be helpful as they provide a useful space to store all relevant material related to the project. It’s important to note that a preregistration protocol does not restrict flexibility. Deviations from the preregistration protocol are normal and usual; they should simply be reported.

## Discussion

The main aim of this study was to analyse the prevalence of transparency and reproducibility-related practices in meta-analyses on the effectiveness of clinical psychological interventions. A random sample of published meta-analyses on the effectiveness of clinical psychological interventions was reviewed. Additionally, the relationship between publication year, preregistration, and guidelines adherence and different indicators was assessed. A lack of transparency in key aspects for the reproducibility of meta-analyses was found.

Regarding preregistration, the 19% of preregistered meta-analyses found in our meta-review is substantially higher than findings from previous studies mainly focused on primary research (Hardwicke, Thibault, et al., 2020b, 3%; Hardwicke, Wallach, et al., 2020c, 0%;) and higher than that found in a previous study focused on meta-analyses (Polanin et al., 2020, 2%). However, the existence of a preregistration was not shown to be associated with an increased reporting of information related to the potential reproducibility of the meta-analysis, except for the specification of the year for first date searched and, to a minor extent, for identification of the estimation method of the heterogeneity variance. The majority of identified preregistrations were allocated in specialized repositories such as PROSPERO, and these were submitted through a structured form. Hence, relevant information, identified in this study as poorly reported, could be explicitly requested, such as: full search strategy, estimation method of the heterogeneity variance, or the formula used to compute the effect measure. As pointed in Table 3, it is worth noting that preregistration is compatible with flexibility, allowing flexibility tracking. Regarding guidelines adherence statements, only 30 of the 100 meta-analyses stated the use of reporting guidelines. Adherence statements to guidelines was associated to higher reporting of the full search strategy, full description of the methods used for assessing risk of bias of individual studies and, to a minor extent, better description of the screening process, coded variables, and the statistics used to compute the effect measure. The suboptimal adherence to many items of PRISMA guidelines have been studied in previous studies (Page & Moher, 2017). An update of PRISMA has recently been published (Page et al., 2021), including new recommendations and changes relevant to some of the aspects examined in this study.

The reporting of search strategy elements in clinical psychology was found to be better than in other areas (Koffel & Rethlefsen, 2016; Maggio et al., 2011; Mullins et al., 2014; Polanin et al., 2020). Nonetheless, there is still room for improvement in aspects such as indicating the limits of the search, specifying search dates or including the full search strategy. Using the same definition, we found the search reproducible in 37% of the meta-analyses, as opposed to the 22% reported in Koffel and Rethlefsen (2016). In any case, the inclusion of a full reproducible search strategy was modest in the set of meta-analyses reviewed. As recommended in Table 2, and in line with the updated PRISMA 2020 (Page et al., 2021), the full search strategy for all databases consulted, detailing dates, limits, specific terms, and the Boolean connectors should be reported. These details could be reported as additional/supplementary information hosted by the journal or third-party repositories.

The validity of a systematic review partially depends on the reliability of the data extraction process. Coding primary studies requires time, attention to details in a tedious task, and multiple choices. Close to one third of the meta-analyses reviewed did not give details on how the study coding process was carried out. In addition, although most of the meta-analyses that reported details of this process carried out double coding, only a third of these reported inter-coder reliability estimates of the coding process. Moreover, missing data is a common problem in evidence synthesis, but only 42% of the meta-analyses reviewed reported any method to deal with missing data. Several methods have been developed to check the robustness of the results to the inclusion of missing data (Mavridis et al., 2014; Pigott, 2019).

Previous studies examined the reproducibility of primary effect sizes of a set of meta-analyses: Gøtzsche et al. (2007) found problems in 37% of these meta-analyses and, Lakens et al. (2017) found significant problems to reproduce a set of meta-analyses, in part due to the lack of information on how the primary effects sizes were calculated and Maassen et al. (2020) found that the main problems with primary effect sizes reproducibility are often related to the ambiguity in the procedure followed by the meta-analyst. Thus, reporting information concerning the primary effects sizes used and their exact and detailed computation methods is essential to reproduce and update a meta-analysis. However, a poor reporting of detailed primary effect sizes computation method was found in our study. As pointed in Table 2, due to the variety of approaches to compute a common kind of effect measure (e.g., *d* index family), more detailed information on this should be specified. Commonly, general references to handbooks have been found, but the specific computation method used should be specified with a mention to the page(s) where the calculation formula(e) can be found. Furthermore, multiplicity of results in primary studies is a common meta-analysis issue and the way to deal with it could have an impact on the meta-analytic model estimates (Maassen et al., 2020; Tendal et al., 2011), but only half of the meta-analyses reviewed reported any method of dealing with it. Also, it is common to find extreme effect sizes in a set of primary studies when carrying out a meta-analysis. Apart from this, the presence of outliers could have an impact on the conclusions, however, only a third of the meta-analyses reviewed dealt with this issue. There are different approaches to handling influential observations such as leave-one-out analyses and Cook’s distances (Viechtbauer, 2010) or graphical examination of heterogeneity using combinatorial meta-analysis (Olkin et al., 2012). Addressing the issue of influential results is a good practice to appraise the robustness of the conclusions derived from the quantitative synthesis.

Regarding synthesis methods, different analytic choices have to be made when a meta-analysis is carried out. As pointed in Table 2, these choices could have an impact on the results (Langan et al., 2015; Sánchez-Meca et al., 2013; Schmidt et al., 2009) and compromise the reproducibility of the meta-analysis and should be reported. However, a lack of transparency was found in the report of relevant information such as the weighting factor used or the estimation method of the heterogeneity variance when a random-effects model was assumed. On the one hand, a comprehensive description of the synthesis methods used in a meta-analysis facilitates the reproducibility, and, on the other hand, it allows the assessment of the robustness of the results when applying different statistical techniques (Steegen et al., 2016). If the meta-analysis is carried out using the R (R Core Team, 2020) package *metafor* (Viechtbauer, 2010), a very helpful function is *reporter()*. This function generates a readable text format output with a draft analysis report based on a previously fitted *rma.uni* object. Such draft may be used as a starting point when writing up the meta-analytic report.

Along with a comprehensive description of the synthesis methods, the availability of open data is the next key aspect that enables the reproducibility of the results as well as checking their robustness. Previous studies found poor ratios of data sharing in primary research in different areas (Alsheikh-Ali et al., 2011; Hardwicke & Ioannidis, 2018a; Hardwicke, Thibault, et al., 2020b; Hardwicke, Wallach, et al., 2020c; Iqbal et al., 2016; Wallach et al., 2018). Despite the majority of the meta-analyses we reviewed having reported at least some raw data, most data were shared in the article itself. Indeed, the vast majority of raw shared data were reported in PDF format, hampering reanalysis attempts by different researchers and most likely forcing them to tedious, time-consuming and, and error-prone manual recoding of the data (Bek, 2019; Wilkinson et al., 2016). Only three studies shared some raw data in interoperable formats such as CSV files. On other hand, the shared raw data were typically limited to the primary effect sizes computed (as opposed to the raw data reported in the primary studies). Conversely, it was uncommon to find primary raw statistics used to compute the effect sizes, similar to previous studies (Polanin et al., 2020). This is the process where more problems have been found to reproduce the results of a meta-analysis (Gøtzsche et al., 2007; Maassen et al., 2020). There is no good reason for a meta-analyst not to share all the coded raw data. We note that, with the exception of individual participant data meta-analysis, the unit of analysis involves summary data from primary studies, hence sharing the meta-analysis database usually entails no ethical concerns. Nowadays, there are many ways for data sharing in interoperable spreadsheet formats, for example hosted by the journal, in online repositories (e.g., OSF, Fighshare, Zenodo), or on personal/institutional webpages. In addition to reproducibility concerns, data sharing allows for quick updating of a meta-analysis and the reusability for new scientific purposes. As mentioned in Table 2, the FAIR principles (Wilkinson et al., 2016) are a useful guideline for best practices in data sharing: meta-analytic data that are findable, accessible, interoperable, and reusable would have a stronger impact and efficiency by decreasing research waste.

Previously, we discussed the relevance of a comprehensive description of synthesis methods to guarantee the reproducibility of the results. However, this form of verbal description is often lacking in detail or contains errors making reproducibility difficult (Hardwicke et al., 2018; Lakens et al., 2017). A better approach to ensure the analytic reproducibility is sharing the analysis script (Hardwicke et al., 2018; Obels et al., 2020), typically in computer code format. Unfortunately, only one meta-analysis shared the analysis script. This result is in line with previous research (Hardwicke, Thibault, et al., 2020b; Hardwicke, Wallach, et al., 2020c; Polanin et al., 2020; Wallach et al., 2018).

Nowadays, there are many options for analysis script sharing, allowing easy reproducibility and detection of potential errors. R (R Core Team, 2020) is a free and open software environment and programming language that, along with RStudio, facilitates the production of easily shared analysis scripts. As noted in Table 2, Moreau and Gamble (2020) share a very useful script template for carrying out a meta-analysis with R using the *metafor* (Viechtbauer, 2010) package in their OSF project: https://osf.io/5nk92/.

The prevalence of funding statements found in our meta-review of meta-analyses of psychological interventions was similar to those reported in the broader fields of psychology (Hardwicke, Thibault, et al., 2020b) and biomedical research (Wallach et al., 2018), and higher than in social sciences research (Hardwicke, Wallach, et al., 2020c). Regarding competing interests, ratios of including a statement were found to be better than for psychology and social sciences research, and similar to biomedical research. Accessibility was fairly adequate compared to biomedical (Wallach et al., 2018) and social sciences (Hardwicke, Wallach, et al., 2020c) research and similar to psychology (Hardwicke, Thibault, et al., 2020b). In any case, there is still room for improvement. Of the 29 meta-analyses for which we could not find any publicly available version, 13 stated that public funding was provided. Public research funders usually have open-access mandates (van Noorden, 2021), which make sense. Green open-access consists of self-archiving a copy of the work in a freely accessible repository (institutional, third-party archive…) or personal webpage and does not entail any extra charge for the authors. Different versions of the manuscript, such as pre-print or an author-accepted version, can be stored.

This study has some limitations. First, the time span covered is fairly wide. Thus, the obtained estimates may not capture the changes that have arisen in recent years. Due our focus on a highly specific area of research our primary goal was to capture general transparency and reproducibility-related practices over a wide time span, and then we subsequently attempted to assess possible variations over time using logistic regression models with publication year as a predictor. Therefore, additional research is needed to examine more specific changes over years. Second, our conclusions might not be generalizable beyond the area of clinical psychology. Additional research is needed to address these issues in different meta-analytic contexts. Third, this study was not preregistered. Although the nature of our analyses is strictly exploratory, there are several benefits of preregistration for all kinds of studies, regardless of their design or aims—mainly regarding transparency in workflow and decision-making processes. We have attempted to address this gap by openly sharing all relevant material at the different stages of the study. Last, our results do not provide findings on the reproducibility of the meta-analyses reviewed, but on the prevalence of transparency and reproducibility-related practices. The reports were reviewed to assess the availability of necessary information and data to be able to check the reproducibility of a meta-analysis. Further research is needed that specifically addresses the analytic reproducibility of published meta-analyses in different research areas.

## Conclusion

Our findings show a relatively better level of transparency and reproducibility-related practices across meta-analyses on the effectiveness of psychological interventions compared to more general fields or research areas. Nevertheless, some gaps were found in key aspects, including full reproducible search, level of detail on statistical methods, availability and interoperability of relevant raw data, and script analysis code sharing. Nowadays, meta-analysis is widely considered as the best source of scientific evidence (e. g., OCEBM Levels of Evidence Working Group, 2011) and therefore meta-analytic results and conclusions often have a strong impact on policymaking, social practices, or healthcare decisions. Thus, standards of research quality, transparency, and reproducibility-related practices of meta-analyses need to be high. Tools to help researchers carry out a meta-analysis with the best open practices are available (e.g., Lakens et al., 2016; Moreau & Gamble, 2020), as well as a recent update of the PRISMA statement (Page et al., 2021). We also provide some recommendations in Table 2 which are particularly relevant to researchers carrying out evidence synthesis in the field of clinical psychology. Increasing compliance to these different recommendation sources will improve the strength of the conclusions of a meta-analysis and will allow a more efficient and stronger development of scientific knowledge. These points are particularly relevant in the context of meta-analytic research recognized and understood as a source of evidence synthesis commonly used to guide applied practice. Flawed meta-analytic conclusions could lead to misguided practical applications, particularly harmful in a healthcare context. Last, this study provides a baseline for comparison that will allow future studies to assess the impact of recent developments in this field.

## Data Availability

All materials, data, and analysis script coded have been made publicly available on the Open Science Framework: https://osf.io/xg97b/. Additionally, a Code Ocean capsule reproducing the reported results is available at: 10.24433/CO.6211364.v1

## Footnotes

1 Only cases that provided data from different sources than the article itself (previously coded in item 48) were re-reviewed. For cases that only provided data in the article itself (item 48 = 2), ‘pdf’ was imputed for each type of data previously coded as available (see script analysis code available at: https://osf.io/a7zth/).
